# In sync or out of sync?

**DOI:** 10.1007/s12471-024-01919-y

**Published:** 2024-12-23

**Authors:** Benoît Delforge, Lukas Spruyt, Becker S. N. Alzand

**Affiliations:** 1 Department of Internal Medicine, UZ Leuven, Leuven, Belgium; 2 Department of Emergency Medicine, UZ Leuven, Leuven, Belgium; 3 Department of Cardiology, AZ Glorieux, Ronse, Belgium

## Answer

This is a sinus bradycardia alternating with junctional escape rhythm. These two different rhythms are independent of each other, and neither is dominant. The sinus rhythm—when conducting—affects the behavior of the junctional rhythm by resetting it, while the junctional rhythm does not influence the sinus rhythm due to the absence of retrograde conduction.

The presence of two different independent rhythms influence each other’s behavior without either being dominant is known as isorhythmic dissociation [1–3]. Occasionally, a period of synchronisation can occur between these two independent rhythms, which is described by the term ‘accrochage’ [4], as observed in our case.

**Fig. 1:**
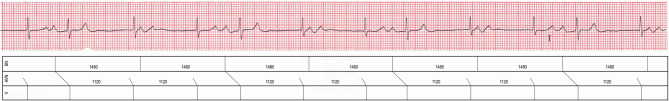
Lead 1 with a ladder diagram showing regular, slow sinus *P* waves unaffected by the junctional rhythm—as there is no retrograde *P* waves—and a regular, faster junctional rhythm being reset by the conducted sinus beats, without influencing its intrinsic rate. *SN* sinus node, *AVN* atrioventricular node, *V* ventricle

The prefix ‘iso-’ is derived from the Greek word ‘isos’ meaning ‘equal’ or ‘same’. In the context of isorhythmic dissociation, ‘iso-’ suggests that the two rhythms within the heart that are separate but similar in some way, such as having approximately the same rate (Fig. 1).
